# Morphological variability of the jugular foramen: a comprehensive anatomical-imaging study emphasizing its compartmentalization

**DOI:** 10.1007/s10143-025-03681-0

**Published:** 2025-06-24

**Authors:** George Triantafyllou, Ioannis Paschopoulos, Panagiotis Papadopoulos-Manolarakis, Nektaria Karangeli, Fotis Demetriou, George Tsakotos, Maria Piagkou

**Affiliations:** 1 Department of Anatomy, School of Medicine, Faculty of Health Sciences, National and Kapodistrian University of Athens, 75 Mikras Asias str, Goudi, Athens, 11527 Greece; 2 Department of Neurosurgery, General Hospital of Nikaia-Piraeus, Athens, Greece

**Keywords:** Jugular foramen, Skull base anatomy, Intrajugular process, Variation, Skull base surgery, Foramen compartmentalization

## Abstract

The jugular foramen (JF) is a structurally complex region of the skull base with critical neurosurgical relevance. This anatomical imaging study aimed to investigate the morphological variability of the JF by analyzing both its intracranial and extracranial orifices using a combination of osteological assessment and computed tomography (CT) scans.A total of 200 adult skulls (100 dried specimens and 100 CT scans) were examined bilaterally. The JF was classified based on the presence, number, and completeness of intrajugular processes (IJPs). Morphometric measurements of horizontal and transverse diameters were performed, and statistical analyses were used to evaluate differences by sex, laterality, and between the intracranial and extracranial aspects.IJPs were significantly more common in the intracranial orifice (41%) compared to the extracranial (19.5%), with type 1a (incomplete septation) being the most frequent. A right-sided predominance was observed for intracranial JF dimensions (*p* = 0.036), while no significant sex-related differences were found. The morphometric analysis revealed that JF compartments became progressively narrower with an increasing number of IJPs. A moderate positive correlation was found between intracranial and extracranial measurements (*B* = + 0.357, *p* < 0.001).This study provides a detailed anatomical and radiological analysis of JF variability, emphasizing its clinical implications for skull base surgery. The findings highlight the importance of preoperative imaging in identifying IJP-related compartmentalization, which may impact surgical planning and risk assessment. Future research should aim to better correlate bony septations with neurovascular content to define their relevance in both surgical and pathological contexts.

**Clinical trial number** Not applicable.

## Introduction

Skull base surgeries often necessitate multiple approaches to target specific regions due to the skull’s complex anatomy. For instance, the jugular foramen (JF) is situated deep within the posterior cranial fossa. It has significant adjacent neurovascular structures, including the internal carotid artery (ICA) anteriorly, facial nerve (VII) laterally, hypoglossal nerve (XII) medially, and vertebral artery inferiorly [16]. Consequently, various surgical approaches, combinations, and variants are employed to access the JF surgically [10].

**Fig. 1:**
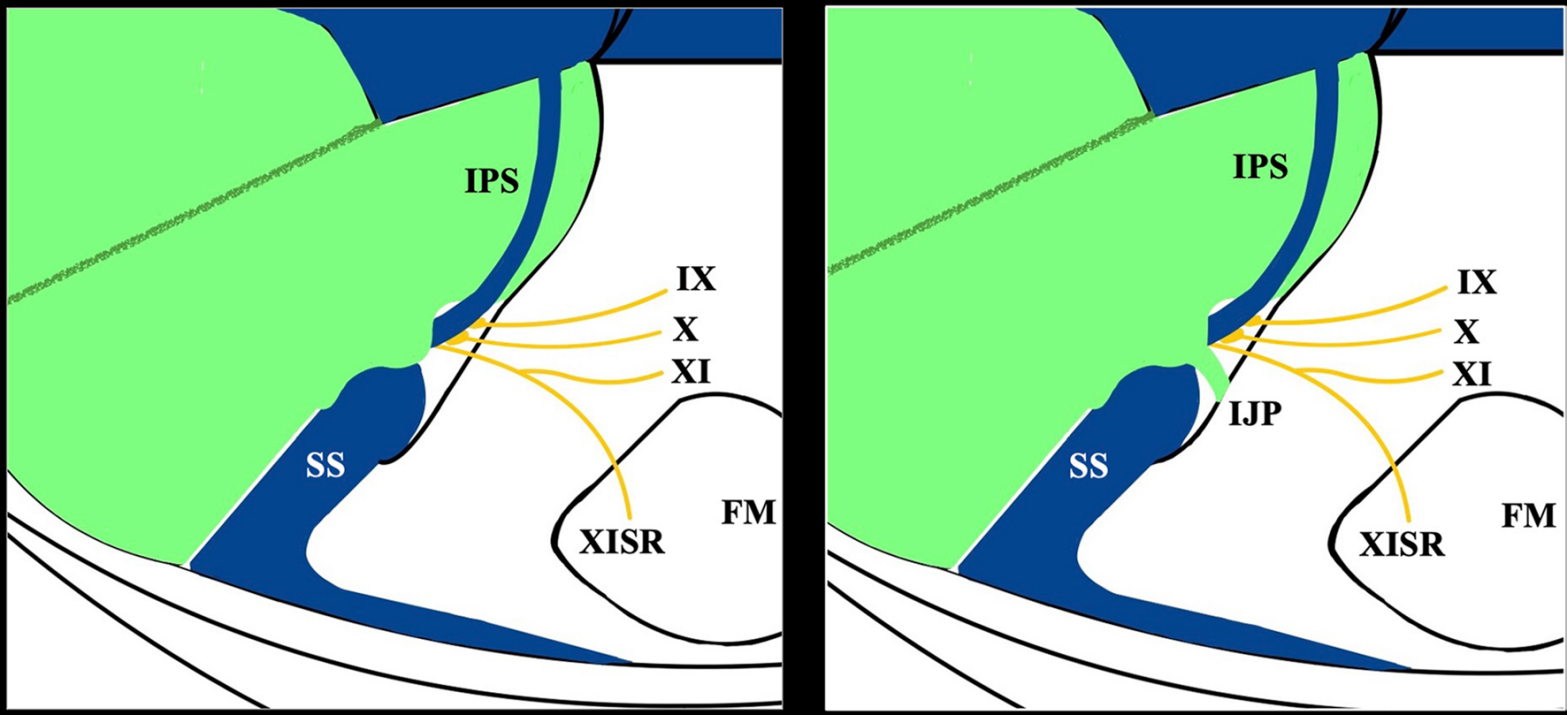
A schematic representation of a typical jugular foramen (JF) and a JF with an intrajugular process (IJP) is presented. SS - sigmoid sinus, IPS - inferior petrosal sinus, and drainage at the internal jugular vein bulb are indicated. The passage of the lower cranial nerves (IX, X, and XI) is also depicted. Additionally, the spinal root of the XI (XISR) enters through the foramen magnum area (FM)

The JF corresponds to a large and irregular hiatus at the petro-occipital junction between the jugular process of the occipital bone and the jugular fossa of the temporal bone. Several critical structures pass through the JF: the inferior petrosal sinus (anterior), glossopharyngeal nerve (IX), vagus nerve (X), and accessory nerve (XI) (middle), as well as the sigmoid sinus (posterior) [19]. Typically, a posterior meningeal branch of the ascending pharyngeal artery traverses through the JF between the vagus (X) and accessory (XI) nerves [2]. However, its presence and morphological variability have been barely described [13]. The intracranial aperture is composed of a posterolateral pars venosa and an anteromedial pars nervosa, which may be distinguished by dural septations or by a fibrous or bony septum [10, 21, 22] (Fig. 1). The bony (intrajugular) processes (IJPs), in the form of septa, are considered to be formed during developmental ossification when the distance between nervous and vascular structures increases [9]. These septa have also been illustrated in fetal specimens [8]. Furthermore, this compartmentalization has undergone extensive study, resulting in numerous theories based on cadaveric microsurgical investigations; however, consensus has yet to be achieved [3].

Studies have shown that JF septation is common and varies among individuals, with some having complete or incomplete bony septations. These variants may contribute to conditions such as JF syndrome or influence cerebrospinal fluid dynamics [16].

Bergman’s Comprehensive Encyclopedia of Human Anatomic Variations elucidates how IJPs divide the JF into distinct parts, most frequently characterized on the intracranial side of the JF [23]. Furthermore, numerous osteological studies have investigated these IJPs [1, 7, 9, 12, 15, 17, 18]. Among these investigations, Fang et al. [9] proposed a notable classification system based on the side of the formation (*petrous or occipital bone*), the number of processes (single or multiple), and completeness. We found the classification system by Fang et al. [9] and the related osteological studies concerning JF morphological variability are particularly compelling, especially in light of the absence of distinct differentiation between the morphologies of the intracranial and extracranial orifices. Therefore, this anatomical-imaging study was designed to provide a detailed evaluation of the morphology of the JF, with particular emphasis on both its intracranial and extracranial orifices. The study also aims to contextualize these findings within the scope of relevant surgical approaches. Our initial hypothesis proposed that the intracranial and extracranial openings of the JF exhibit distinct morphological features, while also exploring the possibility of a measurable correlation between their respective morphometric dimensions.

## Materials and methods

A total of two hundred adult skulls (100 male and 100 female) with a mean age of 48.9 ± 15.1 years were bilaterally evaluated for their JF morphology. One hundred adult dried skulls from the osteological collection of the Anatomy Department at the School of Medicine, National and Kapodistrian University of Athens, were derived from the “Body Donation Program.” [4]. Furthermore, one hundred (100) computed tomography (CT) scans (*slice thickness: 0.4–0.6 mm*) were retrospectively analyzed by the Neurosurgery Department at the *General Hospital of Nikaia-Piraeus* after receiving ethical approval from the relevant authorities (*56485/13.11.2024*). The scans were conducted employing a helical high-speed, low-dose scanner (*SOMATOM go.Top*,* 128-slice configuration*,* Siemens*), with the patient’s head positioned in a supine neutral orientation and subsequently documented utilizing Horos software version 3.3.6 (*Horos Project*). Evidence was gathered from the multiplanar reconstruction of the axial, coronal, and sagittal slices, along with their three-dimensional volume reconstruction. The inclusion criteria mandated high-quality scans and the absence of pathological processes that could distort the area’s anatomy, consistent with previous studies.

**Fig. 2:**
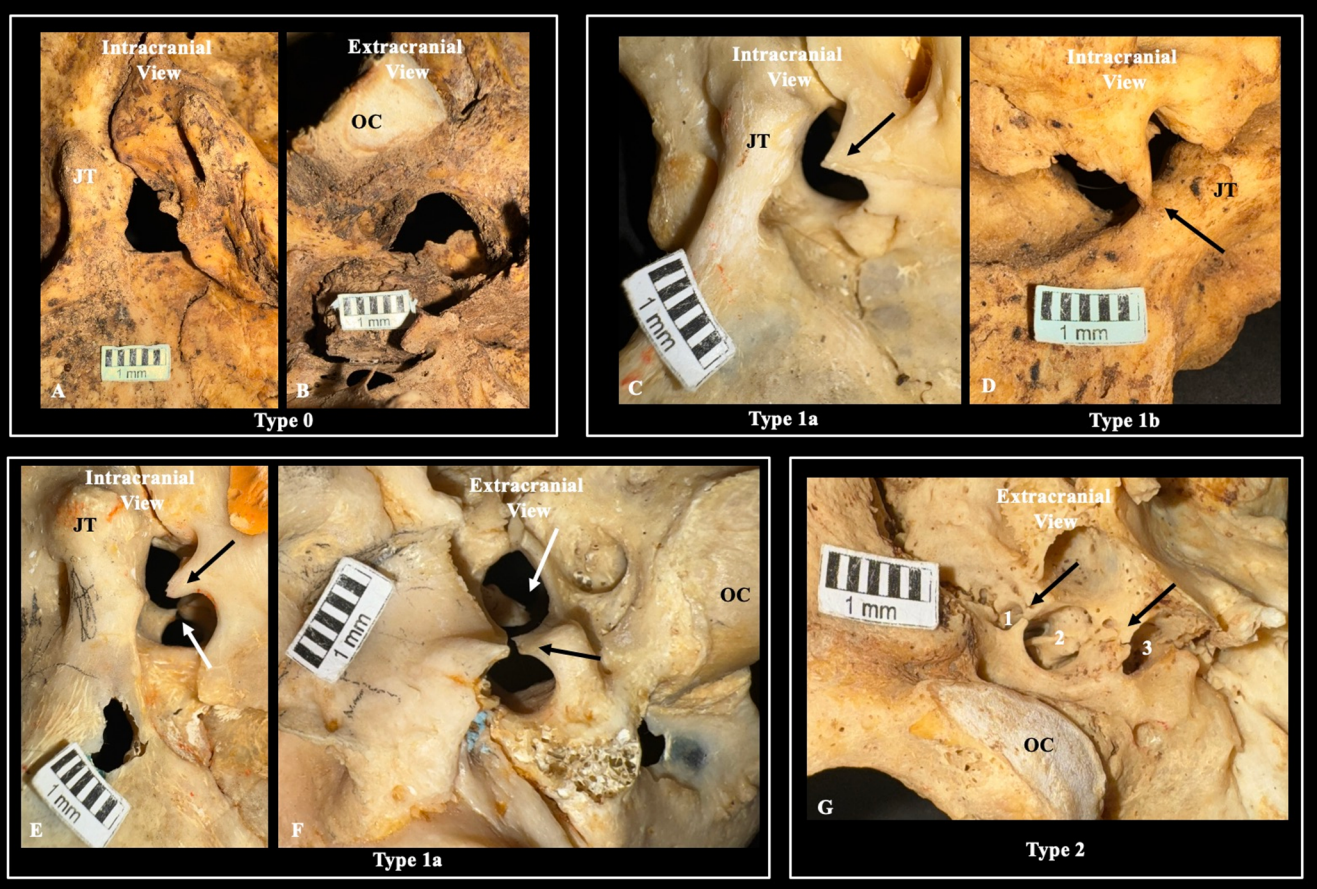
The morphology of the jugular foramen (JF) in dried skulls, both extracranially and intracranially. Type 0 - no intrajugular process (IJP); Type 1a - incomplete formation of IJP; Type 1b - complete formation of IJP. Note the figures in the left corner depicting Type 1a in both extracranial and intracranial orifices (indicated with white and black arrows). Type 2 - two IJPs forming three compartments (1, 2, 3) within the JF. OC - occipital condyles, JT - jugular tubercle

**Fig. 3:**
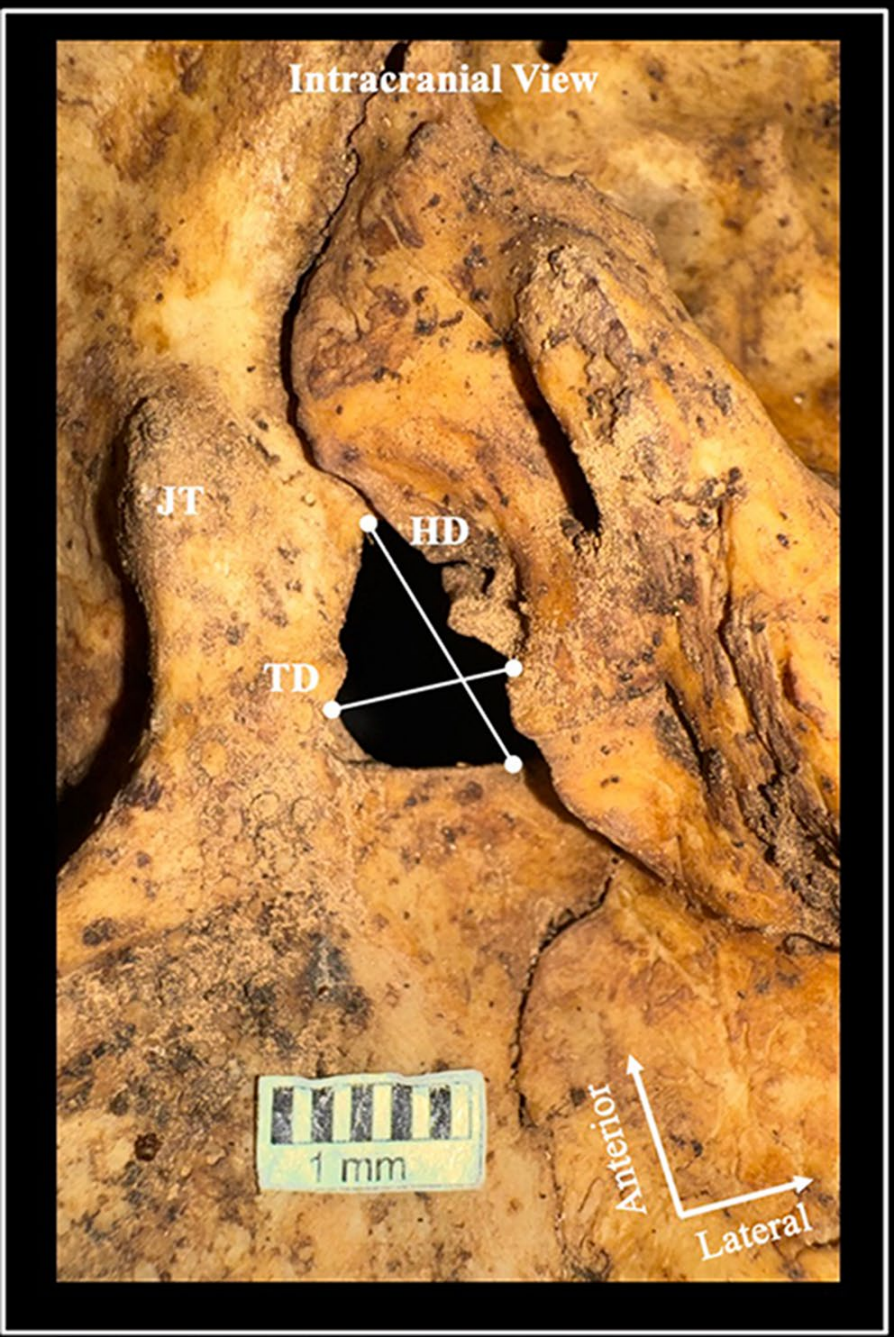
The jugular foramen (JF) morphometric details. HD- maximum horizontal diameter, and TD- maximum transverse diameter. JT- jugular tubercle

The JF was evaluated separately for the presence of IJPs concerning its intracranial and extracranial orifices. A type 0 JF was designated when no IJP was observed (i.e.,* JF of a single compartment*) (Fig. 2). A type 1 JF corresponded to a single IJP (i.e.,* a two-compartment JF)*,* and a type 2 JF was identified when two IJPs were present (i.e.*,* a three-compartment JF*). Type 1 JF had two subtypes based on whether the IJP was incomplete (type 1a) or complete (type 1b) (Fig. 2). The JF morphometry was calculated by measuring the maximum horizontal and transverse diameters (Fig. 3). Morphometric measurements were also obtained for the JF compartments, with measurements taken for each compartment when IJPs were present.

Cohen’s kappa (κ) and the Intraclass Correlation Coefficient (ICC) were utilized to ensure reliability and accuracy in the process. Two independent reviewers (GTr and IP) assessed the sample. In cases of discrepancies, the assessment provided by the senior author (MP) was considered definitive for categorical variables. The mean value of the two investigators’ measurements was calculated for continuous variables. Cohen’s kappa was employed to assess inter-rater agreement for categorical data, with a κ-value of 0.60 or higher interpreted as indicating moderate agreement. For continuous data, the ICC was computed to evaluate consistency between raters, with a value of 0.75 representing good agreement.

Statistical analysis was conducted using IBM SPSS Statistics for MacOS, Version 29 (*IBM Corp.*,* Armonk*,* New York*,* United States*). Nominal data from unpaired observations were compared using the Chi-square test, while McNemar’s test was applied for paired observations. The Shapiro-Wilk test evaluated normality. Continuous variables were analyzed according to measurement type: unpaired measurements were examined with an independent t-test if normality was met; otherwise, the Mann-Whitney U test was used. Results are presented as means and standard deviations unless otherwise specified. A p-value of less than 0.05 was considered statistically significant.

## Results

The JF was bilaterally observed (400/400 sides, 100%). Type 0 JF (single compartment, no IJP) was identified in 322 sides extracranially (80.5%) and in 236 sides (59%) intracranially. The concomitant absence of IJP (intracranially and extracranially) was noted on 178 sides (44.5%). An IJP was recorded on 164 sides intracranially (41%) and 78 sides extracranially (19.5%). Bilateral presence of IJP was identified in 34 skulls (34/200; 17%). Subtype 1a (one incomplete IJP) was the most prevalent, with frequencies of 31% intracranially and 13.5% extracranially. Subtype 1b (one complete IJP) was identified at 6% intracranially and 4.25% extracranially. Type 2 JF (two IJPs) was observed at 4% intracranially and 1.75% extracranially. Overall, the presence of IJP was significantly more frequent in the intracranial than in the extracranial orifice (*p* < 0.001). The results are summarized in Tables 1 and 2 based on their side and sex distribution. Regarding the morphology of the JF by sex, intracranially, males demonstrate a greater frequency of IJPs, particularly subtypes 1a and 1b, which indicates more complex bony septation. A male predominance was noted for the presence of IJP in the intracranial orifice (*p* < 0.001) (Table 1). In contrast, females more frequently exhibit JF of type 0 morphology (uniseptate). Extracranially, both sexes primarily present with JF of type 0, yet again, males more often display complex types (1a, 1b, 2). This observation corroborates the study’s conclusion of a significant male predominance regarding intracranial IJP presence (*p* < 0.001).

**Table 1:** Morphological variability of the intracranial aspect of the jugular foramen (JF) intrajugular processes (IJPs)

JF Intracranial Morphology	Total (*n* = 400)	Left (*n* = 200)	Right (*n* = 200)	*p*-value	Males (*n* = 200)	Females (*n* = 200)	*p*-value
Type 0	236 (59%)	112 (56%)	124 (62%)	0.122	96 (48%)	140 (70%)	< 0.001*
Subtype 1a	124 (31%)	70 (35%)	54 (27%)		84 (42%)	40 (20%)	
Subtype 1b	24 (6%)	7 (3.5%)	17 (8.5%)		16 (8%)	8 (4%)	
Type 2	16 (4%)	11 (5.5%)	5 (2.5%)		4 (2%)	12 (6%)	

**Table 2:** Morphological variability of the extracranial aspect of the jugular foramen (JF) intrajugular processes (IJPs)

JF Extracranial Morphology	Total (*n* = 400)	Left (*n* = 200)	Right (*n* = 200)	*p*-value	Males (*n* = 200)	Females (*n* = 200)	*p*-value
Type 0	322 (80.5%)	162 (81%)	160 (80%)	0.118	153 (76.5%)	168 (84%)	0.102
Subtype 1a	54 (13.5%)	30 (15%)	24 (12%)		29 (14.5%)	26 (13%)	
Subtype 1b	17 (4.25%)	5 (2.5%)	12 (6%)		9 (4.5%)	8 (4%)	
Type 2	7 (1.75%)	3 (1.5%)	4 (2%)		2 (1%)	5 (2.5%)	

**Fig. 4:**
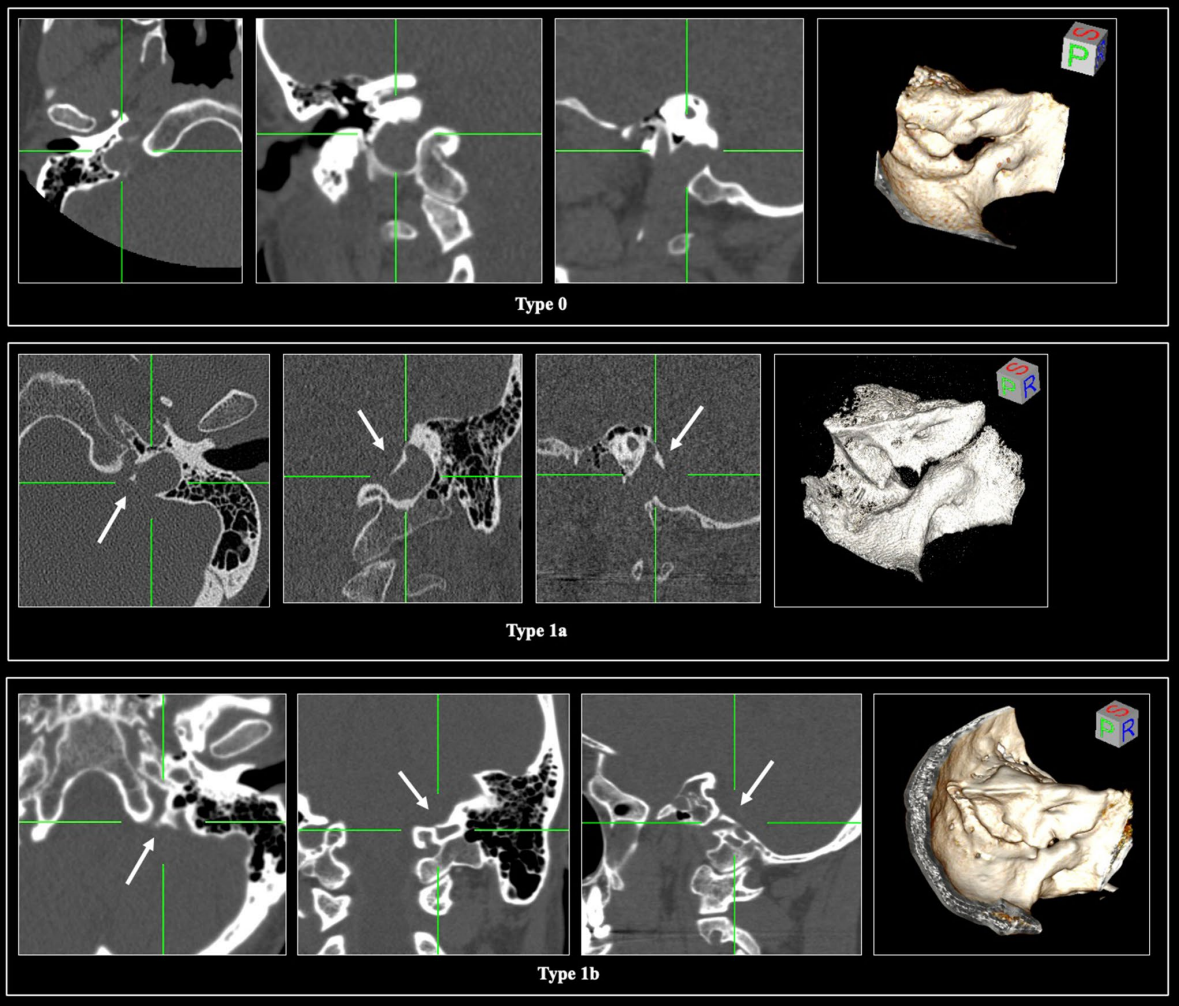
The morphology of the jugular foramen (JF) using computed tomography scans. The presence of the intrajugular process (IJP), classified as type 1, is clearly depicted in axial, coronal, and sagittal slices based on its morphology, whether partial (type-1a) or complete (type-1b). In contrast, the absence of the IJP is designated as type 0

Each type of IJP is illustrated on the osteological specimens in Fig. 2 and the CT scans in Fig. 4. It is important to emphasize that the IJPs are represented in axial, sagittal, and coronal sections (Fig. 4). Cohen’s kappa test coefficient was 0.897, indicating an almost perfect agreement between investigators for the categorical variables.

Regarding the morphometric measurements, the ICC was 0.91, indicating excellent reliability, and the measurements obtained between the different techniques were not significant (*p* = 0.449); therefore, they were merged. The JF compartments formed by the IJPs were significantly narrower according to the number of septations (Tables 3 and 4). This observation was consistent for both intracranial and extracranial orifices (Tables 3 and 4). Intracranially, the JF had larger dimensions on the right side compared to the left, regardless of the JF type (*p* = 0.036). This observation did not hold for the extracranial dimensions (*p* = 0.080). The specimen’s sex did not influence dimensions either intracranially (*p* = 0.699) or extracranially (*p* = 0.552). Nevertheless, a moderate positive correlation was identified when comparing the intracranial and extracranial dimensions (B = + 0.357, *p* < 0.001). Additionally, the extracranial transverse distance had a moderate positive correlation with the extracranial horizontal distance (B = + 0.318, *p* < 0.001). However, this correlation was not observed for the intracranial dimensions (B = + 0.065, *p* = 0.277).

**Table 3:** Morphometric parameters of the intracranial aspect of the jugular foramen (JF) based on its morphology. The results are summarized as mean (Standard Deviation)

JF Intracranial Morphology	Type 0 One compartment (uni-compartmental JF)	Type 1 Two compartments (bi-compartmental JF)	Type 2 Three compartments (three-compartmental JF)	*p*-value
Transverse distance	5.72 (2.10)	3.83 (1.57)	1.84 (0.90)	< 0.001*
Horizontal distance	9.37 (3.30)	4.75 (1.75)	2.65 (1.12)	< 0.001*

**Table 4:** Morphometric parameters of the intracranial jugular foramen based on its morphology. The results are summarized as mean (Standard Deviation)

JF Extracranial Morphology	Type 0 One compartment (uni-compartmental JF)	Type 1 Two compartments (bi-compartmental JF)	Type 2 Three compartments (three-compartmental JF)	*p*-value
Transverse distance	6.48 (2.13)	3.22 (1.46)	1.59 (0.69)	< 0.001*
Horizontal distance	10.97 (3.04)	4.32 (1.45)	3.50 (1.54)	< 0.001*

## Discussion

The current anatomical imaging study evaluated the JF morphological variability by investigating extracranial and intracranial orifices and their potential combinations. We classified the IJPs into types based on their number (type 0, type 1, and type 2) and morphology (incomplete—type 1a and complete—type 1b). The morphometric analysis indicated that the JF compartments narrow as the IJPs increase. The JF extracranial and intracranial dimensions were positively correlated; however, a right-sided predominance was observed in the intracranial dimensions of the JF. Intracranially, IJPs are more frequent and complex, particularly in males. Extracranially, most JFs are uniseptate (Type 0), exhibiting less variation and lacking significant sex or side differences. The notable differences in intracranial morphology by sex suggest the presence of developmental or biomechanical factors that influence septation.

Dodo [8] was among the first to describe the IJPs, presenting anterior and posterior bony septa. Athavale [1] classified IJPs based on location (*anterior or posterior*), number of septa (*one*,* two*,* or multiple*), and degree of ossification (*partial or complete*). He reported a higher frequency of bony septation on the intracranial aspect compared to the extracranial aspect, particularly in males. Consistent with our findings, IJPs were more frequently observed intracranially (41%) than extracranially (19.5%), and males demonstrated greater septation, especially subtypes 1a and 1b. Our classification system further refines this by subdividing Type 1 (incomplete vs. complete) and providing statistical analysis based on side and sex. Fang et al. [9] developed a simplified IJP classification (*complete/incomplete*,* petrosal/occipital origin*,* and one or multiple IJPs*), reporting an 8.5% prevalence in their sample. Our study reports significantly higher frequencies (up to 41% intracranially), likely due to the use of both osteological and high-resolution CT data, as well as the analysis of both intracranial and extracranial aspects. Fang et al. [9] didn’t differentiate between orifice levels, which our study highlights as anatomically and surgically significant. Tekdemir et al. [20] used axial CT Sect. (1 mm slice thickness) to describe JF septation, highlighting the challenge of identifying subtle bony septa. Our study overcomes this limitation by utilizing multiplanar reconstructions and 0.4–0.6 mm CT slices, revealing finer and more frequent septations, particularly incomplete IJPs (Subtype 1a), which may have been overlooked in older imaging protocols. Skrzat et al. [18] noted that osseous bridges were rare, with significant variation in JF dimensions. Emphasized the need for imaging in assessing septation. Our study confirms morphometric narrowing of JF compartments with increasing septation, supported statistically (Tables 3 and 4). The use of 3DCT provides a more detailed assessment compared to the limited resolution in Skrzat’s morphometric data. Rodrigues et al. [15] found right-side predominance in JF size and observed male dominance in horizontal dimensions. Our data similarly reveal right-sided JF enlargement intracranially (*p* = 0.036), but there is no significant sex difference in size. We attributed the right-sided dominance to larger venous structures, consistent with Rodrigues’ observations. Das et al. (2016) noted variability in JF shape and emphasized the clinical relevance in skull base surgery. Our findings extend this by correlating septation with surgical risk (e.g., Vernet’s syndrome) and emphasizing that bony IJPs complicate transjugular approaches. They also link narrow JF compartments to potential venous outflow obstruction, which previous studies hinted at but did not statistically correlate.

The JF is a crucial neurovascular conduit at the skull base, and its variability affects anatomical understanding and clinical practice in skull base and neurosurgical procedures. In our study, JF had significantly wider transverse and horizontal diameters than types with IJPs, both intracranially and extracranially. Intracranially, type 0 had mean transverse and horizontal diameters of 5.72 mm and 9.37 mm, respectively. Extracranially, the corresponding dimensions were 6.48 mm and 10.97 mm, respectively. As the number of IJPs increased (from type 1a to 1b to type 2), the compartment dimensions progressively narrowed, a statistically significant pattern (*p* < 0.001). A right-sided dominance in intracranial JF dimensions was observed (*p* = 0.036), possibly related to venous asymmetry (e.g., dominant right sigmoid sinus). No significant sex-based differences in morphometric values were found, which may contrast with some prior osteological reports. These findings support the “canal-like” hypothesis of the JF, suggesting that it functions as a conduit with distinct and independently variable intracranial and extracranial orifices, rather than as a simple bony aperture.

Skrzat et al. [18] noted wide ranges for JF dimensions: horizontal diameter from 7.8 mm to 23.1 mm, transverse diameter from 3.6 mm to 16.6 mm. These broader ranges likely reflect heterogeneous sample sources and a potential lack of IJP-based subdivision. Our data offer more refined categorization by IJP type, thereby linking morphometry to anatomical compartmentalization, which Skrzat’s study did not explore.

Athavale (2010) indicated that JF with bony septations had smaller overall diameters, suggesting that the formation of IJPs during development leads to narrowed passageways. This conclusion aligns closely with our morphometric findings, where increased septation (type 1 or 2) correlated with significantly reduced JF dimensions.

Tekdemir et al. [20] used CT imaging to describe variations in shape but lacked precise morphometric measurements. Our application of 0.4–0.6 mm CT slices and multiplanar reconstructions provides a more quantitative and reliable assessment compared to earlier imaging-based studies.

Rodrigues et al. [15] found that the horizontal JF diameter in dry skulls ranged from 5.0 to 13.0 mm, with a tendency towards right-sided dominance and larger dimensions in males. Our study confirms the right-sided predominance but found no significant sex difference, possibly due to methodological differences (combined CT and osteology vs. dry skulls only). This right-sided predominance may be attributed to the larger dimensions of the veins (sigmoid sinus, inferior petrosal sinus, and internal jugular vein) on the right side [18, 23]. A recent study identified that 70% of patients had a right-sided dominant sigmoid sinus, which resulted in larger and higher jugular bulbs [14]. Therefore, larger dimensions may be attributed to asymmetric or high jugular bulbs [18, 24]. Nevertheless, reports of asymmetry with right-sided predominance were also noted for the inferior petrosal sinus [26].

However, neither study assessed JF intracranial and extracranial dimensions, which we found to be significantly different from those in our current study. Previous studies evaluating JF morphology separately for its orifices propose that the JF is more likely a canal-like structure than a foramen [20]. This conclusion stemmed from observing distinct morphologies and dimensions of extracranial and intracranial orifices. The study found significant differences in morphology and morphometry, with the intracranial side showing IJPs more frequently, while the extracranial side had larger dimensions. Future studies investigating the complex three-dimensional anatomy of the JF using micro-CT will further enhance our understanding of the theory of the “canal-like” morphology of the JF. In contrast, magnetic resonance imaging (MRI) studies will provide additional details about the structures passing through the JF.

Extensive research on JF compartmentalization has been driven by its surgical implications. Various approaches can access the JF region, including *postauricular transtemporal*,* retrosigmoid*,* far lateral*,* and preauricular infratemporal methods*. *Lateral approaches through the mastoid process* are commonly used in conjunction with mastoidectomy for lesions extending through the JF. For intracranial lesions in the JF, *the retrosigmoid approach* and *extensive far-lateral* and *transcondylar modifications* are used. *The classical retrosigmoid approach* provides access to the cerebellopontine angle and the intracranial JF orifice. Still, it may not be practical due to lesion extension through the foramen magnum (FM) or clivus. *The preauricular infratemporal approach* allows access to the anterior JF, enhancing lateral techniques for anterior lesion extensions [16]. The IJP can impact all these approaches, particularly the transjugular, which may require intraoperative drilling for better visualization. Manipulating neurovascular structures during dissection is complex when encountering a bony septum instead of a fibrous or dural one; thus, there is an increased risk of injury [9].

The presence of IJPs raises the risk of compression syndromes, such as Vernet’s syndrome. Compression affecting cranial nerves IX, X, and XI results in loss of taste, paralysis of the vocal cords, and weakness in the trapezius and sternocleidomastoid muscles. Tumors located in the JF can cause three types of symptoms based on the affected structures: pulsatile tinnitus (*due to proximity to vascular structures*), hearing loss (*if they extend to the middle ear*), and cranial nerve dysfunction (*affecting CN IX*,* X*,* XI*,* and sometimes XII if they extend to the hypoglossal canal*). A thorough physical examination of the patient can provide crucial information about which cranial nerves have been implicated [11]. Additionally, diagnosis should involve MRI of the brain with contrast, along with further examinations such as angiographies (for vascular lesions), a CT of the skull base (bony erosion), or laryngoscopy (vocal cord palsy). Although tumors, inflammation, or injury can cause these syndromes [11], the presence of IJPs significantly narrows the JF compartments, as demonstrated by the current study (Tables 3 and 4). Moreover, we can hypothesize that multiple factors mentioned earlier could contribute to compression syndromes. For example, patients with the presence of IJPs would be more vulnerable to compression if a tumor grows into the JF. Interestingly, Hou et al. [11] noted that stenosis caused by the IJP can lead to restricted venous outflow, increased intracranial pressure, and potentially idiopathic intracranial hypertension. This reduction in the available space for the veins due to the IJPs could justify these issues. Additionally, these pathologies are influenced by the angulation of the dural venous sinuses in the posterior cranial fossa [5, 6].

Another clinically significant anatomical insight regarding the JF is the discovery of lymphatic connections traversing this region. In a pivotal study, Yağmurlu et al. [25] used cadaveric dissection and immunohistochemical techniques to identify a novel anatomical feature: the presence of lymphatic vessels within the JF that establish continuity between deep cervical lymph nodes and intracranial lymphatic pathways. This finding supports the hypothesis that the JF may act as a potential conduit for the bidirectional spread of malignant cells between the central nervous system and peripheral lymphatic systems.

This study presents several limitations that merit consideration. Although the sample size (*n* = 400 sides) was adequate for the morphometric analysis of the JF, a larger and more geographically diverse sample would likely enhance the detection of rarer variants, thereby improving generalizability. Additionally, all specimens were obtained from a single regional population (Athens, Greece), which may introduce population-specific anatomical biases. From a methodological perspective, the use of CT scans with a slice thickness of 0.4–0.6 mm may lead to the misclassification of incomplete IJPs, particularly when narrow gaps (< 0.5 mm) are falsely interpreted as complete osseous septa. Furthermore, the reliance on osteological and radiological materials precluded the inclusion of soft-tissue or neurovascular elements, limiting the study’s ability to correlate bony structures with the actual contents of the JF compartments.

Future research utilizing high-resolution micro-CT or MRI, along with cadaveric specimens that preserve neurovascular and lymphatic structures, is necessary to correlate bony prominences with functional compartmentalization, particularly in the context of surgical planning and pathophysiological modeling.

## Conclusions

This anatomical imaging study provides a comprehensive evaluation of the morphological variability of the JF, with a particular emphasis on distinguishing between its intracranial and extracranial orifices. By classifying IJPs based on their number and morphology, the study enhances our understanding of the anatomical complexity and compartmentalization of the JF. Quantitative morphometric analysis demonstrated that increasing numbers of IJPs are associated with progressive narrowing of JF compartments, a feature of critical relevance to surgical access and risk stratification. Additionally, the observed positive correlation between intracranial and extracranial dimensions supports the concept of the JF functioning as a canal-like conduit rather than a discrete foramen, aligning with emerging anatomical theories. These findings collectively underscore the importance of high-resolution preoperative imaging to detect anatomical variants that may influence the choice of transjugular and lateral skull base approaches. Accurate delineation of bony structures, particularly IJPs, can aid in minimizing neurovascular complications and improving surgical outcomes.

## Data Availability

No datasets were generated or analysed during the current study.
